# EHMTI-0374. Gene-based pleiotropy across migraine with and migraine without aura

**DOI:** 10.1186/1129-2377-15-S1-H2

**Published:** 2014-09-18

**Authors:** D Nyholt, H Zhao, B de Vries, E Eising, A van den Maagdenberg

**Affiliations:** 1 Genetics and Computational Biology, QIMR Berghofer Medical Research Institute, Brisbane, Australia; 2 Department of Human Genetics, Leiden University Medical Centre, Leiden, Netherlands; 3 Departments of Human Genetics and Neurology, Leiden University Medical Centre, Leiden, Netherlands

## Introduction

There has been extensive discussion in the migraine field concerning whether migraine with aura (MA) and migraine without aura (MO) are distinct subtypes or part of the same disease spectrum.

## Aims

Utilizing a novel gene-based (statistical) approach, we aimed to identify specific genes and pathways associated with both MA and MO.

## Methods

Gene-based tests were performed utilizing genome-wide association summary statistics results from the recent International Headache Genetics Consortium (IHGC) study comparing 5,118 MA cases to 74,239 controls, and 7,107 MO cases to 69,427 controls. After accounting for non-independence of gene-based test results, we examined the significance of the proportion of genes associated across MA and MO.

## Results

We found a highly significant overlap in genes associated with MA and MO. For example, of the total 1,297 genes with a nominally significant gene-based p-value (Pgene-based ≤ 0.05) in the MA subgroup, 132 genes also produced Pgene-based≤ 0.05 in the MO subgroup. The proportion of overlapping genes is almost double the empirically derived null expectation, producing highly significant evidence of gene-based overlap (Pbinomial-test= 8.36 × 10-10) between both migraine subtypes.

The genes overlapping MA and MO were enriched (P < 0.05 and Fold enrichment > 1.2) for molecular functions of 'actin binding', 'phosphatase activity', 'growth factor activity', 'protein homodimerization activity', and 'protein dimerization activity'; and were overrepresented in networks related to 'Auditory and Vestibular System Development and Function', 'Cellular Development', and 'Cellular Growth and Proliferation'.

## Conclusions

Our results provide important insight into the likely genes and biological mechanisms underlying MA and MO.

No conflict of interest.

